# Draft Genome Sequence of a Mycobacterium heckeshornense Clinical Isolate

**DOI:** 10.1128/genomeA.00178-18

**Published:** 2018-03-29

**Authors:** V. Ustinova, T. Smirnova, D. Varlamov, Y. Monakhova, E. Larionova, S. Andreevskaya, I. Andrievskaya, L. Chernousova, A. Ergeshov

**Affiliations:** a Microbiology Department, Central Tuberculosis Research Institute RAMS, Moscow, Russia; b All-Russia Research Institute of Agricultural Biotechnology, Moscow, Russia; c Syntol, LLC, Moscow, Russia

## Abstract

We report here the draft genome sequence of Mycobacterium heckeshornense, isolated from the sputum of a patient admitted to a tuberculosis hospital with suspected pulmonary tuberculosis.

## GENOME ANNOUNCEMENT

Mycobacterium heckeshornense was given a species status in 2000 by Andreas Roth and coauthors (1). They isolated a culture of acid-fast M. xenopi-like bacteria from bronchoalveolar lavage, sputum, and surgical samples of a Heckeshorn Lung Clinic (Berlin, Germany) patient with lung disease. 16S rRNA gene sequencing revealed that the culture belonged to a new species, M. heckeshornense. Although cases of M. heckeshornense isolation from patients with mycobacteriosis are rare, there have been several published articles describing clinically significant cases of mycobacteriosis affecting bones (2, 3), lymph nodes (4), and lungs (1, 5). We report here the draft genome sequence of M. heckeshornense, isolated from the sputum of a patient admitted to a tuberculosis hospital with suspected pulmonary tuberculosis.

An X ray revealed changes in the lung, but all sputum specimens obtained from the patient did not contain acid-fast bacilli. In August 2011, a resection of the affected part of the lung was performed. Microscopy of the lung tissue sample stained with fluorescent dyes showed the presence of a large number of acid-fast bacilli that grew on a Middlebrook 7H9 liquid medium in the Bactec MGIT 960 system (Becton, Dickinson, USA). The resulting culture consisted of acid-fast bacteria according to the results of microscopy. Species identification of the culture with the GenoType CM/AS test system (Hain Lifescience, Germany) gave no result. The 16S rRNA gene sequencing revealed the organism to be a strain of M. heckeshornense.

The genomic DNA of M. heckeshornense was extracted by the guanidinium thiocyanate DNA isolation method with sorption on magnetic beads and purified by phenol-chloroform-isoamyl alcohol separation followed by ethanol precipitation. For sequencing, two types of libraries were prepared using the Nextera XT DNA library prep kit and the Nextera mate-pair library prep kit. Sequencing was performed on the Illumina MiSeq platform with 2 × 250 paired-end MiSeq V2 chemistry. The obtained reads were analyzed and quality-checked using FastQC version 0.11.3 (6) and subsequently trimmed; Illumina adapters, bases with quality scores lower than Q30, N bases, and reads shorter than 50 bp were removed using Trimmomatic version 0.33 (7). The genome was assembled using SPAdes version 3.9.0 (8). The quality of the assembly was analyzed with QUAST (9). The final assembly consisted of 65 contigs comprising 4,824,707 bp with an *N*_50_ value of 204,041 bp and a GC content of 65.94%.

The M. heckeshornense strain CTRI-134 genome was annotated using the NCBI Prokaryotic Genome Annotation Pipeline (PGAP) (10). It contained 4,725 genes in total, with 4,417 protein-coding genes, 257 pseudogenes, 3 rRNA-coding sequences (5S, 16S, and 23S rRNAs), 45 tRNAs, and 3 noncoding RNAs. Two clustered regularly interspaced short palindromic repeat (CRISPR) arrays were found in the genome.

### Accession number(s).

This whole-genome shotgun project has been deposited at DDBJ/ENA/GenBank under the accession number MPJF00000000. The version described in this paper is the second version, MPJF02000000.
